# Inherited lung cancer: a review

**DOI:** 10.3332/ecancer.2020.1008

**Published:** 2020-01-29

**Authors:** Viviane Teixeira Loiola de Alencar, Maria Nirvana Formiga, Vladmir Cláudio Cordeiro de Lima

**Affiliations:** AC Camargo Cancer Center, R Prof Antônio Prudente, 211 São Paulo, SP 01509-010, Brazil

**Keywords:** hereditary cancer, lung cancer, germline mutation

## Abstract

Lung cancer is the most common cancer worldwide and has high rates of mortality. The major risk factor associated with this disease is tobacco smoke, but approximately 10%–25% of all lung cancer cases occur in patients who have never smoked. Data suggest that lung cancer in never-smokers has a different molecular profile, tumour microenvironment and epidemiology than that in smokers. Several risk factors have been associated with its occurrence, and the possibility of inherited predisposition is becoming clearer. A better understanding of this disease is essential for the future development of personalised screening, diagnosis and treatment approaches, with consequent reduction of mortality. In this review, we discuss historical studies of lung cancer in never-smokers and the currently available evidence of inherited predisposition to this disease.

## Introduction

Lung cancer is the most commonly diagnosed cancer (accounting for 11.6% of all cases) and the leading cause of cancer death (accounting for 18.4% of all such deaths) in both sexes worldwide; it is responsible for more than 1.8 million deaths each year [1]. The high mortality rate associated with this disease is attributable in part to its diagnosis at advanced stages, with regional or distant spread, in approximately 80% of cases; at such stages, treatment is less effective and survival rates are considerably lower (5-year survival, 57.4% in early stage versus 5.2% with distant metastasis at diagnosis) [2].

Incidence rates of lung cancer vary among regions as a reflection of different historic patterns of tobacco exposure, which includes exposure intensity and duration, cigarette type and degree of inhalation and the evolution of these patterns over time, commonly called the ‘tobacco epidemic’. Recent statistics [1] show that incidence rates among men are the highest in Micronesia/Polynesia, Eastern Asia and much of Europe, especially Eastern Europe. In Africa, the rates remain generally low, although they are intermediate to high in several countries in the northern and southern regions of the continent, notably in Morocco (31.9/100,000) and South Africa (28.2/100,000). Among women, incidence rates are highest in North America, Northern and Western Europe and Australia/New Zealand. Interestingly, the rate among Chinese women is similar to those among women in several Western European countries, such as France, despite substantial differences in smoking prevalence between these populations. This rate may be explained by continued exposure to smoke from charcoal burned for heating and cooking purposes [1].

The two major histological forms of lung cancer are non-small cell lung cancer (NSCLC), which accounts for about 85% of cases, and small cell lung cancer (SCLC). NSCLC can be categorised into three subtypes: squamous cell carcinoma, adenocarcinoma and large-cell carcinoma [3]. Several environmental risk factors are associated with its development, including smoking, passive smoking, air pollution, pulmonary diseases (i.e., tuberculosis, chronic bronchitis), occupational exposure to carcinogenic chemicals, ionising radiation (i.e., radon decay products and x-rays), asbestos and alcohol consumption [4, 5]. Tobacco smoking, responsible for more than 80% of all lung cancer cases in Western populations, increases the relative risk (RR) of lung cancer 10–20-fold in smokers compared with never-smokers [1, 6], with its incidence correlated in a dose–response manner with the cumulative amount and duration of tobacco smoked [7]. Smoking is linked most strongly to SCLC and squamous cell carcinoma, whereas adenocarcinoma is the most common type of lung cancer in patients who have never smoked [3].

According to global statistics, however, only about 10%–15% of all smokers develop lung cancer [8], and 10%–25% of all lung cancers are not attributable to smoking [9, 10]. The latter percentage is even higher, up to 30%–40%, in Asian countries [10]. If considered separately, lung cancer in never-smokers (those consuming <100 cigarettes/lifetime) would rank as the seventh most common cause of cancer death worldwide, before cancers of the cervix, pancreas and prostate [11]. Environmental tobacco smoke exposure (passive smoking) is a known risk factor in some of these cases, as it has been demonstrated to increase the risk of lung cancer development in non-smokers by at least 20%. Passive smoking, however, is associated with only approximately 16%–24% of lung cancer cases in non-smokers [12]. Today, we know that lung cancers in smokers and never-smokers are two distinct entities, which differ not only epidemiologically but also in their molecular alteration profiles and tumoral microenvironment compositions.

Lung cancer in never-smokers is diagnosed most commonly in women. Patients tend to be younger, respond better to treatment and have better prognoses, due to the more frequent occurrence of actionable mutations that enable specific treatments with receptor tyrosine kinase inhibitors. For example, *ALK* rearrangements are more common in non-smokers with adenocarcinoma than in smokers with adenocarcinoma or squamous cell carcinoma [13]. Metabolic syndrome also has been linked to the development of lung adenocarcinoma, especially in never-smokers, as genome-wide association studies (GWASs) have demonstrated that *EGFR*,* VTL1A*,* TNFRSF10C*,* C3ORF21* and hyper-methylations of *TNFSF10C*,* BHLHB5* and *BOLL* are involved in both pathways [13, 14].

Tumoral microenvironments also appear to be distinct in smokers and never-smokers, as tobacco smoking causes DNA damage in lung epithelial cells and alters the local immune system [13]. In 2018, Li and colleagues [15] performed a study based on 11 lung cancer microarray datasets with samples from 1,111 lung adenocarcinomas and 200 adjacent normal tissues. They found that the pathways involved in carcinogenesis differ in smokers and never-smokers due to differences in outcomes related to mast cells and CD4+ memory T cells, and their active or resting status. Resting mast cells were associated with better prognoses, and their number was found to be decreased in tumour samples relative to those from adjacent normal tissue. On the other hand, activated mast cells (macrophages) and activated CD4+ memory T cells were associated with poor prognoses and were found in greater numbers in tumour samples. The authors observed more resting mast cells and CD4+ memory T cells, both associated with better prognoses, in tissue samples from never-smokers, and more activated cells of these types, correlated with worse prognoses, in samples from smokers [15].

With the current application of policies to control tobacco use worldwide, the proportion of lung cancer cases in never-smokers is expected to increase. Thus, a better understanding of the disease’s causal factors, which include hereditary predisposition, is needed.

The essential role of the early detection of lung cancer in the survival rate is well established [2]. Since the publication in 2011 of The National Lung Screening Trial Research [16], which showed a 20% reduction in mortality from lung cancer with the use of low-dose computed tomography (CT) for the screening of individuals at high risk of the disease, the screening of current and former smokers aged 55–74 years with at least 30 pack-year smoking histories has been recommended [17]. The development of multiple primary malignancies associated with lung cancer has been linked to a hereditary factor and should also be included in more specific patient follow-up protocols [18].

Squamous cell carcinoma, which is usually more prevalent among smokers, also may have a genetic trigger in non-smokers. In 2017, to better understand tumour growth, Park and colleagues [19] analysed the genetic profile of squamous cell carcinoma in non-smokers using comparative genomic hybridisation arrays. They found that the proto-oncogene *GAB2* (11q14.1) was frequently amplified in these patients and was likely to contribute to disease development [19].

With a better understanding of which non-smokers are at greater risk for lung cancer (e.g., those with inherited susceptibility alleles), the development and application of broader screening guidelines could impact the survival of these individuals. Better elucidation of the physiopathology of these cancers may lead to the development of additional preventive strategies and more accurate approaches to treatment, possibly with new therapeutic targets.

## Familial aggregation in lung cancer

Familial clustering of cancer is characterised by the occurrence of the same type of cancer in two or more first-degree relatives, which may be attributable to inheritable gene mutations. The clinical expression of the disorder is influenced by hereditary predisposition, variable gene penetrance and environmental factors [4]. We estimate today that 5%–10% of all cancers are caused by inherited germline mutations, many of which are associated with known hereditary cancer syndromes [20].

Familial aggregation is the clustering of a disease within families, which may be attributable to genetics, environmental causes or exposure to infectious agents. It is a necessary, although not sufficient, condition for the inference of genetic susceptibility, and studies assessing it are usually first steps in the identification of hereditary diseases. The inherent idea is to determine whether an individual’s risk of a disease is enhanced by the existence of relatives with the same illness. It may be inferred by comparing the incidence of a given disease among relatives of affected subjects and in a suitable control group, typically the relatives of healthy subjects [21, 22]. Familial clustering of cancer may be explained by shared environmental factors, inherited mutation of moderate- and high-penetrance genes and/or inherited single nucleotide polymorphisms (SNPs). GWASs, for example, are effective for the detection of common alleles that contribute to the inherited components of common cancers. To assess the genetic effect, many researchers calculate the ratio of the observed incidence of cancer among first-degree relatives to the expected frequency, called lambda (*λ*).

The literature contains several important studies of familial aggregation that has raised the inferred possibility of genetic susceptibility to lung cancer [5, 22]. The first studies to suggest a genetic component for lung cancer were the Baltimore and Buffalo studies performed by Tokuhata and Lilienfeld [23, 24]. They found a 2.5-fold greater risk of mortality among smoking first-degree relatives of lung cancer probands than among smoking relatives of controls. The Baltimore study [18] also showed that familial aggregation for lung cancer existed with or without the influence of smoking, although the risk of lung cancer development was higher among smokers.

Two studies of familial clustering that have provided important information about specific cancers were based on the Utah family database, from the Utah cancer registry study, which included approximately 125,000 individuals between 1952 and 1992 [25], and the Swedish cancer registry, which included 1,283,047 cancer cases between 1958 and 1997 [26]. In the Utah study, the familial risk of lung cancer was significantly high [*λ* = 2.55, 95% confidence interval (CI) 2.08–3.08]. The study identified significant familial clustering among tobacco-related cancers, such as cancers of the lung, larynx, lip and cervix, which was attributable to shared environmental factors potentially associated with genetic susceptibility. The authors also reported a greater RR for female relatives of female probands than for male relatives of male probands [11, 19]. The Swedish study showed that the risk of lung cancer in first-degree relatives of 88,589 lung cancer cases was increased by 1.9 times (95% CI 1.6–2.4), similar to that of other common cancers with well-known genetic components, including breast (*λ* = 1.5) and colon (*λ* = 1.9) cancers. This type of study does not enable determination of whether the clustering is due to genetic or environmental factors, but the environmental influence was minimised in the Swedish cohort, which included individuals from different generations and thus with different exposures [26].

Another study was performed using data from the Icelandic Cancer Registry on all 2,756 patients in the Icelandic population diagnosed with lung carcinoma between 1955 and 2002 [27]. The familial risk was greater among parents (*λ* = 2.69, 95% CI 2.20–3.23), siblings (*λ* = 2.02, 95% CI 1.77–2.23) and children (*λ* = 1.96, 95% CI 1.53–2.39), and lesser among second-degree (*λ* = 1.4) and third-degree (*λ* = 1.2) relatives, although it was significantly higher than expected in the latter groups. For all groups of relatives analysed, the risk of lung cancer was greater for relatives of patients with early-onset disease [27].

More recently, Cannon-Albright *et al* [28] published the results of another analysis based on the Utah population database, performed to estimate individuals’ RRs of lung cancer based on their family histories of the disease. In contrast to the Iceland study, which involved the estimation of risks based on population rates of lung cancer, the researchers used rates of individuals with no family history of this malignancy. They identified a total of 5,048 lung cancer cases and estimated that individuals with any family history of lung cancer were more than twice as likely to be diagnosed with lung cancer as were those with no family history. RRs were significant for first-, second- and third-degree relatives. For first-degree relatives, they ranged from 2.57 (for one or more such relatives) to 4.24 (for three or more such relatives). For second-degree relatives, in the absence of first-degree relatives, RRs ranged from 1.41 (for one or more such relatives) to 4.76 (for four or more such relatives). For third-degree relatives, in the absence of affected first- and second-degree relatives, RRs ranged from 1.18 (for one or more such relatives) to 1.55 (for four or more such relatives). The authors also found a greater risk associated with a first-degree relative’s diagnosis at an earlier age (50–60 years). A limitation of this study is that data on tobacco use were not available.

In 2018, Ding and colleagues [29] performed an observational study of the clinicopathological characteristics of 152 patients with the familial lung cancer on the Yunnan-Guizhou Plateau in China. Unique characteristics of these subjects were earlier disease onset; greater frequency of cancer among women, with the most frequent histology of adenocarcinoma; and the tendency to have other cancer histories and present with distant metastasis, consistent with poor prognoses. These findings are controversial when considered together with those of the study on tumour microenvironments and prognosis mentioned above [15].

To better validate whether the familial pattern of a disease is consistent with a genetic model of inheritance due to the existence of a major predisposition gene, a different type of study, such as a segregation analysis, is needed [12]. Two studies [30, 31] have investigated the probability of inheritance of a major gene for lung cancer predisposition, and both showed that the pattern of lung cancer occurrence in families is consistent with Mendelian co-dominant inheritance of a rare autosomal gene. Sellers *et al* [30] estimated that segregation at this presumed lung cancer predisposition locus could be responsible for 69% of lung cancer cases at the age of 50 years, 47% at the age of 60 years and 22% at the age of 70 years, possibly as a reflection of an increasing proportion of tobacco exposure-related lung cancer in non-carriers [17, 30]. These studies were important to raise the possibility of a genetic predisposition to lung cancer and to point to the need for further analysis to identify a candidate gene.

## Inheritable predisposition genes associated with lung cancer

### Candidate genes associated with lung cancer

In recent decades, many studies have been performed to gather evidence of a genetic predisposition to lung cancer. Findings from more than 1,000 candidate gene studies have been published in the past 25 years, as such studies are cost effective and relatively easy to perform.

A meta-analysis of 1,018 publications describing 2,910 variants in 754 genes and loci was conducted to interpret genetic associations of common variants with lung cancer [32]. Credibility assessment of the accumulated epidemiological evidence from 246 eligible variants suggested that eight genetic variants (APEX1 rs1760944, AXIN2 rs2240308, CHRNA3 rs6495309, CXCR2 rs1126579, CYP2E1 rs6413432, HYKK rs931794, PON1 rs662 and REV3L rs462779) were strongly associated with the risk of lung cancer development, and ten (ATM rs189037, CD3EAP rs967591, CYP2A6 rs1801272, HIF1A rs11549467, PDCD5 rs1862214, PROM1 rs2240688, TP53 rs12951053, TP63 rs10937405, WWOX CNV-67048 and XRCC1 rs3213255) were moderately associated with this risk. An analysis based on the histological type of cancer revealed significant associations for 25 variants in the NSCLC group, eight of which (CHRNA5 rs16969968, CLPTM1L rs402710, CYP2E1 rs6413432, ERCC1 rs11615, FGFR4 rs351855, HYKK rs931794, MIR146A rs2910164 and TERT rs2736098) demonstrated strong cumulative epidemiological evidence. Five variants in the SCLC group showed significant associations, but these associations were moderate (CHRNA5 rs16969968, CYP1A1 rs4646903 and NQO1 (rs1800566) and weak (GSTM1 present/null and XPC rs2228001) [32], which may point to a greater contribution of genetic predisposition in patients with NSCLC than in those with SCLC.

A candidate gene study published in 2017 employed germline sequence data from 555 lung adenocarcinoma cases from The Cancer Genome Atlas to evaluate possible risks associated with the ATM serine/threonine kinase gene (*ATM*), the *BRCA2* DNA repair-associated gene (*BRCA2*), the checkpoint kinase 2 gene (*CHEK2*), *EGFR*, the parkin RBR E3 ubiquitin protein ligase gene (*PARK2*), the telomerase reverse transcriptase gene (*TERT*), the tumour protein p53 gene (*TP53*) and the YES-associated protein 1 gene (*YAP1*). They found 14 pathogenic mutations in five genes with a frequency of 2.5%. These mutations occurred more frequently in genes associated with the DNA repair pathway, such as *ATM* (50%), followed by *TP53*,* BRCA2*,* EGFR* and *PARK2* [33].

## GWASs

More recently, GWASs have been developed as alternatives to candidate gene studies, as they provide broader coverage of genetic variation through the genotyping of up to 1,000,000 genetic variants or SNPs [5]. They are highly powered to enable the identification of common risk alleles, which are usually of low penetrance [12]. Three GWASs for lung cancer [from the International Agency for Research on Cancer (IARC), MD Anderson Cancer Center, and deCODE Genetics], published in 2008, identified the same locus in chromosome region 15q25, an area that includes a cluster of nicotinic acetylcholine receptor genes (*CHRNA3, CHRNA5 and CHRNB4)* and is associated strongly with lung cancer [34–36]. The IARC study was later expanded, leading to the discovery of an additional susceptibility locus on chromosome 5p, in a region that includes *TERT* and the cleft lip and palate transmembrane 1-like gene (*CLPTMIL*) [37]. The IARC and MD Anderson studies linked these genetic regions directly to lung cancer risk, and the deCODE study identified a sequence variant in the cluster of nAChR genes on chromosome 15 that was associated with smoking quantity, and thus indirectly to elevated cancer risk [34–36]. In a GWAS reported in 2019, Hung *et al* [38] detected three SNPs (rs31490, rs380286 and rs4975616), also mapped to the 5p15.33 CLPTM1-like TERT region, which were linked to genetic susceptibility to lung cancer in a European cohort of never-smokers.

The association between 15q25 and lung cancer seems to be strong with an 80% increase in the risk of lung cancer development for individuals who inherit two alleles [5], which is probably not due solely to the influence of tobacco. In 2009, findings of an analysis of Chinese two-stage case–control studies (3,500 lung cancer cases and 3,300 controls) were published [39]. The three alleles identified in the studies of Caucasian populations mentioned above [34–36] were found to be rare in the Asian population, but the study demonstrated an increased risk of lung cancer associated with four other SNPs in 15q25 (rs2036534C > T, rs667282C > T, rs12910984G > A and rs6495309T > C). This effect was similar among never-smokers and ever-smokers and was independent of sex [39].

In 2014, a GWAS of data from more than 20,000 cases and 40,000 controls revealed a strong association of the risk of squamous cell carcinoma development with rare variants of BRCA2-K3326X [rs11571833; odds ratio (OR) = 2.47, *p* = 4.74 × 10^-20^] and CHEK2-I157 (rs17879961; OR = 0.38, *p* = 1.27 × 10^-13^) [40]. For adenocarcinoma histology, the authors described a common variation at 3q28 (TP63; rs13314271; OR = 1.13, *p* = 7.22 × 10^-10^) previously associated with cancer only among Asians.

A study published in 2018 showed that chromosome 15q25 modifies lung cancer risk and is involved in the pathogenesis of this disease via activation of the neuroactive ligand receptor interaction pathway. This pathway, which is also associated with neuropsychiatric disorders, congenital diseases and nicotine dependence, consists mainly of a group of neuroreceptor genes and is involved in environmental information processing and signalling molecules and interactions [41].

Other variants associated with lung cancer susceptibility in GWASs are 6p21 and 5p15 [42, 43]. A meta-analysis of 16 GWASs including approximately 14,900 lung cancer cases and 29,485 controls of European descent identified risk loci at 5p15, 6p21 and 15q25 with histology-specific effects for the first two variants [44]. The researchers also identified a locus for squamous cell carcinoma susceptibility at 9p21 [44].

### Linkage analysis studies

As GWASs tend to identify common risk alleles of low penetrance, based on the common disease–common variant hypothesis (i.e., that a small number of common risk alleles confer small to moderate risks of disease), they have explained only a small portion of the hereditary risk of lung cancer [45]. With the aim of identifying high-penetrance genes with stronger effects, linkage analyses have also been performed. These analyses are based on the rationale that some genetic traits are attributable to genes that are linked, and thus inherited together. This type of study helps to identify whether a disease phenotype is caused by the mutation in one gene alone, or whether mutations in other genes may be responsible for a similar or identical phenotype [46]. In 2004, Bailey-Wilson *et al* [47] published a genome-wide linkage study, performed using an autosomal-dominant model, of 52 families of probands with lung cancer who had several first-degree relatives with the same disease; they identified a major susceptibility locus related to lung cancer risk in the 6q23–25 chromosomic region. This study proved that significant gene linkage for familial lung cancer exists, suggesting that some rare genetic variants play important roles in lung cancer susceptibility.

### Sanger sequencing and whole-genome sequencing

Based on these findings, another hypothesis becomes appealing: the common disease–rare variant hypothesis, according to which risk loci contain multiple rare independent risk alleles across populations, each with moderate to high penetrance (high allelic heterogeneity). To explore this hypothesis, direct sequencing is the better option, as it enables the identification of all rare alleles in a population. Sanger sequencing, also known as ‘first-generation sequencing’, was the first technique used to sequence the human genome. It is still used widely for the sequencing of individual genes, but, due to its high cost, ‘second-generation sequencing’ or next-generation sequencing (NGS) with multi-gene panels is now performed [45]. NGS has been adopted widely and can be used to sequence more target genes with less DNA and reduced cost, time and labour than required for Sanger sequencing.

In the following paragraphs, we explore the main genes that are possibly related to hereditary lung cancer, discovered in studies based on these two techniques.

## Genes associated with inherited lung cancer syndromes

### EGFR T790M

Case reports have described germline mutations in the kinase domain of the *EGFR*, such as R776G, R776H, T790M, V843I and P848L, which confer a greater risk of cancer development. Among these, T790M seems to be associated with a specific lung cancer syndrome targeting never-smokers [48]. In various studies, Sanger sequencing has correlated familial clustering of lung cancer with a germline T790M mutation in *EGFR* [49–56]. This mutation is rare, but *EGFR* is considered to be a major cancer predisposition gene, associated with an estimated 31% risk of lung cancer development in non-smoking carriers [52]. Although the presence of the *EGFR* T790M mutation seems to be sufficient to induce tumour formation in mouse models [56], this mutation is characterised as weakly oncogenic. When associated with a common activating EGFR mutation, however, the oncogenic potential is significantly enhanced, possibly contributing to earlier disease onset. Seventy-three percent of patients with lung cancer and the germline T790M mutation have been found to carry a second activating mutation [51], most commonly the L858R mutation [49].

The germline T790M *EGFR* mutation is present in approximately 1% of NSCLC cases [52] with a median age at diagnosis of 40 years, and the most common histology is adenocarcinoma. Although somatic mutations in *EGFR* are more common among patients from East Asia, the prevalence of germline mutations in lung cancer cases in Japan was lower than that in North America [51], and, to our knowledge, no case of germline T790M *EGFR* mutation in East Asia has been reported in the literature. Sporadic *EGFR* mutation and germline T790M *EGFR* mutation are associated more frequently with female sex and non-smoking status. The most frequent presentation in CT scans is bilateral ground-glass opacities and pulmonary nodules with an indolent disease course [51]. The screening of unselected populations has no proven benefit, but, as the germline T790M *EGFR* mutation is present in approximately 50% of patients with baseline T790M *EGFR* identified in pre-treatment evaluation [52], some authors have suggested that these patients should be referred for routine germline genotyping to identify carriers [42, 44].

Recently, the Dana-Farber Institute performed the INHERIT *EGFR* Study, which investigated families with this variant. Among the 105 participants, germline *EGFR* mutations were found in 63% of patients with *EGFR* T790M detected in lung cancer tissue at diagnosis, and in 62% (16 of 27) and 44% (4 of 9) of first- and second-degree relatives of germline carriers, respectively. The affected individuals had a median age at lung cancer diagnosis of 57 (range 28–82) years; 81% were white and 19% were black; 52% were never-smokers; 65% of cases were diagnosed at advanced stage (IV); and imaging analysis suggested an indolent multifocal nodular phase, progressing to lymph-node and then remote metastatic disease. Tumour genotyping revealed somatic *EGFR* co-mutations in 94% of probands: 6 exon 19 del, 12 L858R, 6 G719X, 1 exon 19 del and G719R, 1 L861Q, 2 H773R and 1 V774M. Patients who were treated with osimertinib presented no unexpected toxicity [57].

### TP53 (Li-Fraumeni syndrome)

Li-Fraumeni Syndrome (LFS) is a rare hereditary condition associated with a germline mutation of *TP53* that predisposes the patient to the occurrence of cancers in multiple organs, usually with early onset. Statistics show that approximately 50% of carriers will develop cancer by the age of 30 years with lifetime risks of up to 70% in men and 100% in women [58].

Tumours classically associated with this syndrome include breast cancer, soft-tissue sarcoma, osteosarcoma, brain tumours and adrenocortical carcinoma. Lung cancer occurs in 2.3%–6.8% of patients with LFS [58], most frequently men, with a median age at diagnosis of 48 years [59]. Lung cancer is not included in the classic LFS criteria (proband diagnosed with sarcoma at <45 years of age, first-degree relative with cancer diagnosed at age <45 years, and first- or second-degree relative with cancer onset at age <45 years or sarcoma at any age). In 2009, an extended version of the Chompret criteria was published, which included lung cancer in the LFS tumour spectrum, as follows: a proband with a single tumour of LFS spectrum (sarcoma, premenopausal breast cancer, brain tumour, adrenocortical, leukaemia or lung [bronchoalveolar] cancer) diagnosed at age <46 years and at least one first-degree or second-degree relative with a tumour of LFS spectrum (except breast cancer if the proband has breast cancer) with onset at age <56 years or with multiple tumours; OR a proband with multiple tumours (except multiple breast cancers), two of which are tumours of LFS spectrum, with the first occurring at age <46 years; OR a proband diagnosed with an adrenocortical carcinoma or choroid plexus carcinoma, irrespective of family history [58].

In 2017, preliminary data from the LIFESCREEN randomised clinical trial revealed a greater frequency of lung adenocarcinoma [22% (five patients, most non-smokers)] among patients with the syndrome [60]. In an analysis of 555 lung adenocarcinoma cases from The Cancer Genome Atlas, 14 pathogenic mutations in five genes were identified. Among patients with *TP53* mutations, the most common variants found were *TP53* R267Q*, TP53* P152L and* TP53* R158L, all previously linked to LFS [33].

In some reports, *EGFR* mutations have been detected in patients with LFS who develop lung cancer; the loss of p53 function may free the promoter of the *EGFR* gene and make the gene more susceptible to the occurrence of mutations [61].

### BRCA

*BRCA* mutations have always been associated more frequently with breast and ovarian cancer syndrome, but a 2017 study showed that the frequencies of other cancers were higher among subjects in hereditary than in non-hereditary branches of the families of patients eligible for *BRCA* testing, especially in the lung, kidney, liver and larynx [62]. Members of hereditary family branches had greater probabilities of lung cancer (OR = 4.5, 95% CI 2.15–9.38), liver cancer (OR = 4.02, 95% CI 1.14–14.15) and laryngeal cancer (OR = 3.4, 95% CI 1.12–10.39), independent of sex and age. These findings are important not only to guide the application preventive measures but also for the future development of possible therapeutic approaches.

### Human epidermal growth factor 2 (HER2)

HER2 is an oncogene in the EGFR family. It is overexpressed in patients with breast cancer at a frequency of 15%–20% [63]. In lung cancer, however, somatic mutation of HER2 is rare. Such mutations are found in 1.6%–2.5% of NSCLC cases [64]. They are more frequent among nonsmokers, adenocarcinomas, patients of Asian ethnicity and females [65] and are associated with poor treatment response [64].

A Japanese study published in 2013 was the first to describe germline HER2 mutations in the transmembrane domain as conferring potential susceptibility to lung cancer [65]. The germline variant G660D was identified through whole-exome sequencing in a Japanese family with a high rate of lung cancer. The proband was a woman who was a light smoker with a history of multiple lung adenocarcinomas at the age of 44 years who was treated with lobectomy, but subsequently recurred. At the time of the study, she was 53 years old and had multiple bilateral lung adenocarcinomas. With the hypothesis that mutations in the transmembrane domain of HER2 act as drivers, the researchers sequenced exon 17 of HER2 in samples of 315 sporadic NSCLCs, 253 of which were adenocarcinomas, and identified a novel non-synonymous somatic mutation, V659E, in one patient. These data, however, are not definitive for the attribution of carcinogenic potential to these mutations, and further studies are needed [66].

### YAP1

YES-associated protein 1 is a transcriptional coactivator that influences tissue growth and organ size. In 2015, a germline mutation (*YAP* R331W) in the transactivation domain was identified by NGS of samples from a family with lung adenocarcinoma clustering and validated in an external cohort of 1,135 participants without cancer and 1,312 patients with lung adenocarcinoma [66]. The proband of the original family was a mother of four affected daughters and one unaffected son. In this family, individuals in whom *YAP1* mutation was detected (smokers and never-smokers, aged 50–89 years) had been diagnosed with lung adenocarcinoma or were being monitored due to the detection of ground-glass opacities. This allele was associated with a 5.9-fold increased risk of lung cancer development [67].

A study published in 2017 demonstrated that YAP acts as an *EGFR* downstream signalling molecule, modulating lung cell proliferation, and is a potential therapeutic target for EGFR-dependent adenocarcinomas [68].

### CHEK2

Kukita *et al* [69] reported on two siblings with histories of multiple primary lung cancers at the age of 60 years. The female patient subsequently developed breast cancer, and the male patient had a history of prostate and colon cancers. Exome analysis revealed non-synonymous homozygous mutations in *CHEK2*,* FCGRT*,* INPP5J*,* MYO18B* and *SFI1*. The mutation in *CHEK2* altered the tertiary structure of CHK2 by disrupting the salt bridge between p.R474 and p.E394, making it unstable. This effect appeared to have a causative relationship with the familial cancer described, as no structural change was observed in the other mutated genes. However, further studies need to be performed to establish the pathogenic role of this *CHEK2* mutation in human carcinogenesis.

## Other genes

Kanwal *et al* [70] analysed germline mutations associated with the familial lung cancer in nine subjects (four patients with lung cancer and five healthy relatives) using whole-genome sequencing. Among the 12 most highly mutated genes, as validated by a polymerase chain reaction and DNA sequencing, they identified five genes (*ARHGEF5*, *ANKRD20A2*, *ZNF595*, *ZNF812* and *MYO18B*) with germline mutations potentially associated with lung cancer development. Some mutations in the *MUC12*, *FOXD4L3* and *FOXD4L5* genes occurred at greater frequencies in samples from subjects with the familial lung cancer or in lung cancer tissue compared with samples from healthy subjects. The authors also found germline mutations in many genes of non-coding RNA.

In a study published in 2018, whole-genome sequencing of samples from 36 never-smoking Chinese patients with lung adenocarcinoma diagnosed at ≤45 years of age identified 4,344–60,259 somatic mutations per tumour [70]. To evaluate genetic predisposition risk, germline variants from the 36 initial patients and 28 additional lung adenocarcinoma cases were analysed; the frequency of pathogenic and likely pathogenic germline mutations among young patients was 78.3%. The main germline mutations identified were *BPIFB1* (rs6141383, p.V284M), *CHD4* (rs74790047, p.D140E), *PARP1* (rs3219145, p.K940R), *NUDT1* (rs4866, p.V83M), *RAD52* (rs4987207, p.S346*) and* MFI2* (rs17129219, p.A559T). A *TP53* missense germline mutation (rs121912664, p. R205H) was found in one individual with a tumour that appeared to be hypermutated relative to the others. As the overall survival of these patients was not altered by the presence of germline mutations, their mutations should not be considered to be predictive of prognosis [71].

## Discussion

Genetic counselling for lung cancer remains a challenge. No guideline for genetic testing has been established based solely on a family history of lung cancer in non-smokers, unless the proband fulfils criteria for a known hereditary syndrome.

In addition, genetic testing options for lung cancer remain very limited. Most commercial laboratories offer limited or no testing for hereditary lung cancer variants, and no guideline has been established for the best way to manage unaffected carriers of variants associated with lung cancer, such as germline *EGFR T790M*, *HER2* and *YAP1* mutations. Screening with low-dose CT may be incorporated into the care of these patients, but the optimum frequency of such evaluation is uncertain.

## Conclusions

Lung cancer in never-smokers is the seventh most lethal cancer worldwide, and its incidence will increase in the upcoming years, as the tobacco burden is falling rapidly in many countries. Much remains to be learned about this pathology, which has proven to be very different from lung cancer related to smoking, not only epidemiologically but also at the molecular level. Many affected patients show clear hereditary predisposition, and several genes have been associated with an increased risk of lung cancer development. As the prognosis is related closely to the disease stage at diagnosis, a better understanding of the genetic factors associated with lung cancer in never-smokers and of when genetic counselling is indicated is needed to provide effective screening methods and detect asymptomatic carriers in predisposed families. More research is warranted to provide evidence-based screening guidelines for carriers of germline lung cancer risk alleles.

## Funding statement

No funding was received for this article.

## Conflicts of interest statement

The authors declare no conflicts of interest.
